# Robust ISAC based framework for location estimation and target detection in 6G networks

**DOI:** 10.1371/journal.pone.0337050

**Published:** 2026-02-12

**Authors:** Lav Soni, Ashu Taneja, Nayef Alqahtani, Jarallah Alqahtani

**Affiliations:** 1 Chitkara University Institute of Engineering and Technology, Chitkara University, Punjab, India; 2 Department of Electrical Engineering, College of Engineering, King Faisal University, Al-Ahsa, Saudi Arabia; 3 Computer Science Department, College of Computer Science and Information Systems, Najran University, Najran, Saudi Arabia; Beijing Institute of Technology, CHINA

## Abstract

To enhance spectrum utilization and situational awareness in sixth generation (6G) networks, integrated sensing and integration (ISAC) is introduced as a unified functionality. This paper proposes a centralized ISAC based framework operating within a Cloud-Radio Access Network (C-RAN) architecture. The system employs multiple transmit and receive access points equipped with uniform linear antenna arrays to enable simultaneous communication and high-resolution environmental sensing. A hybrid signal transmission model is developed, incorporating both line-of-sight (LoS) and non-line-of-sight (NLoS) channels under realistic propagation conditions. Time of Arrival (TOA), Time Difference of Arrival (TDOA), and Direction of Arrival (DOA) techniques are implemented for cooperative localization, while radar-based target detection is analyzed using hypothesis testing. The localization mean square error (MSE) and probability of detection (*P*_*D*_) are evaluated for different number of receivers *M* and number of observation samples *L* under varied signal-to-noise ratio (SNR) values. It is observed that a gain of 8.75dB is achieved at SNR of 10dB with DOA estimation as the value of *M* is changed from 5 to 10. Also, the *P*_*D*_ improves with increasing *M* and *L* offering a gain of 15 dB with Swerling model-1 and 20 dB with Swerling model-2. The impact of noise standard deviation $\sigma_{d}$ and $\sigma_{\phi }$ on the estimation accuracy is also presented. In the end, it is shown that the proposed ISAC framework offers scalable solutions for 6G IoT networks and autonomous systems with enhanced localization accuracy and detection reliability.

## 1 Introduction

### 1.1 Need and motivation

Wireless communication technology has evolved significantly, leading to data-driven fifth-generation (5G) networks that offer high data rates, low latency, and enhanced connectivity [1,2]. As research progresses toward sixth-generation (6G) systems expected around 2030, the focus extends beyond high rate communication to high resolution sensing through shared spectrum and hardware. 6G combines communication and sensing into a single wireless infrastructure to provide intelligent, immersive, and context-aware applications [3]. This deep integration, termed Integrated Sensing and Communication (ISAC), is going to back 6G technology [4]. Unlike previous generations that focused solely on enabling human-to-human or machine-to-machine data exchange, 6G envisions a cyber-physical ecosystem where the wireless network not only facilitates communication but also acts as a ubiquitous, high-resolution radar-like sensor [5]. This means that 6G base stations (BSs), user devices, and access points (APs) will be capable of simultaneously sensing their environment and transmitting data over the same frequency spectrum [6]. This convergence blurs the lines between traditional communication and radar systems, enabling a wide range of new capabilities such as precise localization, object tracking, activity recognition, and environmental mapping [7].

### 1.2 ISAC frameworks

The sensing-assisted communication (SAC) framework enhances the reliability of wireless links by embedding radar-like sensing functions, which significantly reduce the overhead of repetitive beam training and channel estimation in high-mobility environments. Previous efforts mostly focused on using sensing capabilities to increase communication efficiency [8]. But in the context of 6G, sensing is seen as an essential service in addition to communication, especially for newly developed environment-aware applications [9]. Researchers have proposed exchanging sensing measurements across dispersed devices or a central fusion node to enable high-precision sensing channels wireless [10]. The paradigm is prevalent in several fields, including linked autonomous cars, over-the-air computation, IoT, and extended reality (XR). For instance, AI-driven edge intelligence transmits large volumes of sensory data between edge nodes and central servers for real-time, accurate perception tasks like object recognition and scene interpretation. Conventional networks rely on dispersed sensors and centralised data fusion, whereas communication aided sensing (CAS) uses device-free wireless sensing inherent in 6G [11]. This enables BSs or RSUs to simultaneously gather and communicate environmental data to end-users, reducing the need for additional sensors. The procedure consists of a sensing process (SP) where the BS records target observations, and a communication process (CP) where users receive the collected sensory data [12]. ISAC systems use the communication-assisted sensing (CAS) architecture to improve connection, environmental awareness, and reduce latency and deployment costs.

### 1.3 Our contribution

The spectrum and hardware overhead owing to increasing data traffic and device density are the main challenges in modern 6G networks. A unified framework integrating sensing and communication has become vital in achieving intelligent, spectrum-efficient and environment-aware connectivity. This paper proposes a centralized ISAC framework to perform joint communication and high-resolution sensing. Through a hybrid signal transmission model and realistic channel characterization, the framework enhances detection reliability, localization accuracy, and spectrum utilization under practical deployment conditions. The main contributions of this work are summarized as follows:

- A centralized ISAC system is designed within a C-RAN environment, integrating multiple transmit and receive APs to enable cooperative multi-static sensing and simultaneous downlink communication.
- The numerical formulations of channel modeling involving communication and sensing signals are provided. The model incorporates both line-of-sight (LoS) and non-line-of-sight (NLoS) propagation environments with spatial correlation, path loss, fading, and interference, providing a realistic and credible evaluation of system performance.
- Three localization methods, namely, Time of Arrival (TOA), Time Difference of Arrival (TDOA), and Direction of Arrival (DOA) are implemented and analyzed for estimating target locations under noise and synchronization uncertainties.
- The localization RMSE is evaluated for different number of receivers under different noise and angular standard deviations
- A radar based target detection model based on hypothesis testing (Neyman–Pearson) is developed to investigate the impact of SNR, antenna diversity, and radar cross-section (RCS) variations on probability of detection, considering realistic target fluctuation models, Swerling models.

## 2 Related work

This section reviews the research work in ISAC systems covering key frameworks, signal processing techniques, performance parameters, reported outcomes and limitations. For example, the authors in [19] highlight that ISAC is vital for future mmWave networks but limited by the energy and sensing capacity of individual base stations (BS). To address this, a multi-BS collaborative sensing approach is proposed, assigning tasks to the most energy-efficient BSs. To avoid excess energy usage, an energy-efficient ISAC scheme using dual-functional radar-communication (DFRC) beams is introduced. The problem is modeled as a mixed-integer nonlinear program, and an EE-CBS algorithm is developed. Simulations show significant energy savings over non-cooperative or non-DFRC methods. The study [20] investigates a cell-free ISAC MIMO system where distributed APs jointly serve users and sense targets. A sensing SNR expression for multi-static sensing is derived, using signals from multiple APs. Two baseline beamforming methods are proposed: one prioritizing communication, the other sensing. Then, a joint sensing and communication (JSC) beamforming design is developed using a max-min fairness approach. The investigation in [21] addresses a more realistic scenario where the communication user and target lie in different fields (near-field and far-field). New beamforming designs are proposed to optimize sensing performance in a bi-static setup by maximizing the sensing signal-to-clutter-plus-noise ratio (SCNR). A generalized iterative algorithm is developed, and results highlight how field mismatch, antenna size, and channel conditions impact sensing performance. The research work in [22] proposes an optimized beamforming and power modulated (BPM) ISAC transceiver design for mmWave 6G systems, addressing the spectrum efficiency (SE) loss caused by limited RF chains and added sensing functions. One RF chain is dedicated to sensing, while the rest support communication using index modulation to maintain SE. An optimization problem is formulated to minimize the sensing beampattern mean squared error (MSE). The study in [23] introduces a two-stage joint localization method that simplifies the angle-of-arrival (AOA) and differential time-delay (DTD) problem by sequentially refining position estimates using hybrid AOA–TDOA and AOA–time delay (TD) measurements, resulting in improved accuracy and robustness compared to constrained weighted least-squares (CWLS) algorithm, particularly when AOA noise is significant. The authors in [24] propose an instant single low earth orbit (LEO) satellite positioning scheme that avoids long trajectory-based measurements by jointly exploiting time–frequency Doppler analysis and beam switching. The method attains 97.5% positioning accuracy within 50 m at 17 dB SNR, comparable to existing approaches.

To provide context for the proposed work, the recent advancements and key developments in ISAC systems are summarized in Table 1. It presents a concise summary of current research directions and their associated contributions. The comparative study including recent Artificial Intelligence (AI) based ISAC models or hybrid Orthogonal Time Frequency Space (OTFS) based detection is presented in Table 2.

**Table 1:** Recent studies covering the advancements and key developments in ISAC systems.

Ref.	System Model	Signal Processing Used	Performance Parameters	Outcomes	Limitations
[13]	OFDM-JSAC	Hybrid Beamforming, Optimization	Accuracy, Efficiency, Throughput	The proposed beamforming schemes enhance detection accuracy, improve efficiency and validate the OFDM-JSC system.	Increased computational complexity, limited real-time implementation.
[14]	MIMO Radar	Optimization, precoder design	Capacity, Efficiency, Mutual Information	The study derives a closed-form sensing mutual information expression and optimizes precoder design via manifold-based methods.	Theoretical assumptions, limited practical applicability.
[15]	Multi-antenna full-duplex	Beamforming, Interference Cancellation, Estimation, Detection	Capacity, Estimation accuracy, Efficiency	The joint full-duplex radar–communication system achieves higher rates and more accurate target estimation than independent operation.	Limited performance due to interference, reduced estimation accuracy.
[16]	MIMO	Estimation, Localization	Accuracy, Rate, Efficiency	The cooperative ISAC scheme improves the average data rate and enables flexible sensing–communication trade-offs.	Performance gain limited by path loss, less effective scaling law.
[17]	OFDM-ISAC	Optimization	Rate	The proposed SCA-based optimization algorithm improves the worst-case communication rate while satisfying power and sensing constraints.	High computational complexity, limited real-time applicability.
[18]	RIS aided ISAC	Localization, Reinforcement Learning	Coverage, Accuracy, Throughput	The proposed framework achieves balanced sensing–communication performance with near-reference accuracy and coverage.	Dependent on precise device locations, degraded performance due to suboptimal placements.

**Table 2:** Comparative study of AI-based ISAC models and hybrid OTFS-based detection.

Ref.	Base	System Model	Application	Benefit	Limitation
[25]	Generative AI	ISAC network	Wireless sensing	Prevents unauthorized sensing, low cost	Added model complexity
[26]	Generative AI	ISAC	Data augmentation	Improves data quantity and quality	Training overhead
[27]	OTFS	OTFS-ISAC	Hybrid beamforming, CSI estimation	Path-loss mitigation, Doppler resilience, improved SKR	High complexity, CSI errors, mobility sensitivity
[28]	OTFS	OTFS-ISAC	Target detection	High accuracy, low complexity	Main-path selection sensitivity
[29]	OTFS	OTFS MIMO ISAC	Multi-target sensing	Reduced RF chains	Performance degrades under fractional delay/Doppler
[30]	OTFS	Cell-free OTFS uplink	Massive random access	Robust to high mobility, wide coverage, low complexity	Fractional parameter resolution sensitivity

## 3 System model

**Fig 1:**
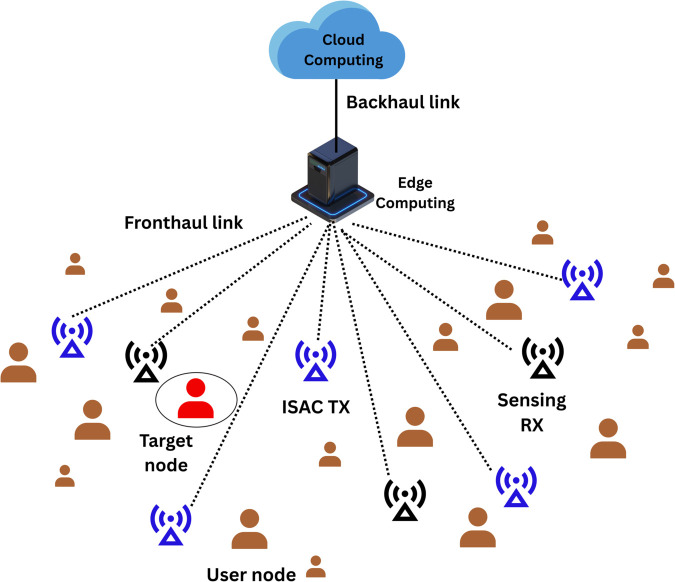
System model integrating sensing and communication.

An ISAC system operating under a centralized Cloud Radio Access Network (C-RAN) architecture is considered with multi-static sensing and downlink communication [31]. Fig 1 shows the system model for the ISAC system. The system consists of *N*_*t*_ transmit access points (APs) and *M* receive APs. Each AP is equipped with *K* isotropic antennas arranged in a uniform linear array (ULA) with half-wavelength spacing. All APs are connected to a central edge cloud via high-speed fronthaul links and are fully synchronized. The system serves *U* user equipments (UEs), each randomly located within a 200m×200m area. The total available system bandwidth is 20 MHz, and each frame lasts 1 ms. In this setting, the *N*_*t*_ transmit APs collaboratively serve the UEs using centrally precoded downlink signals. Let us suppose, a communication symbol for UE *u* be denoted by *s*_*u*_ such that $E{\left\{|s_{u}|\right\}}^{2}=1$ with zero mean and unit power. Also, sensing symbol be denoted by *s*_0_ with zero mean and unit power satisfying $E{\left\{|s_{0}|\right\}}^{2}=1$. It is assumed that sensing signals are independent of the UE data signals. The transmitted signal vector at AP *q* and time slot *τ* is expressed as:

$${𝐱}_{q}[\tau ]=\sum_{u=0}^{U}\sqrt{\rho_{u}}{𝐰}_{u,q}s_{u}[\tau ]={𝐖}_{q}{𝐃}_{s}[\tau ]\rho ,$$ (1)

where $s_{0}[\tau ]$ represents the sensing symbol, $s_{u}[\tau ]$ the communication symbol for UE *u*, $\rho_{u}$ the power control coefficient for UE symbol *u*, and ${𝐰}_{u,q}\in {\mathbb{C}}^{K}$ is the precoding vector for AP *q* associated with symbol *s*_*u*_. The matrix ${𝐖}_{q}=[{𝐰}_{0,q},\ldots ,{𝐰}_{U,q}]$ and ${𝐃}_{s}[\tau ]=diag(s_{0}[\tau ],s_{1}[\tau ],\ldots ,s_{K}[\tau ])$, $\rho =[\sqrt{\rho_{0}},\sqrt{\rho_{1}}.....\sqrt{\rho_{U}}{]}^{T}$. Each AP’s transmit power is constrained to a maximum of $P_{\text{tx}}=30$ dBm (1W), such that:

$$P_{q}=\sum_{u=0}^{U}\rho_{u}\;𝔼[\|{𝐰}_{u,q}{\|}^{2}]\leq P_{\text{tx}}$$ (2)

### 3.1 Channel modeling

### 3.2 Communication channel model

A block fading model is used where the channel remains constant within a coherence time and changes independently across blocks. The channel vector from transmit AP *q* to UE *u* is denoted as

$${𝐡}_{u,q}\sim CN(0,{𝐑}_{u,q})$$ (3)

where ${𝐑}_{u,q}$ is the spatial correlation matrix capturing the effects of path loss, shadowing, and antenna correlation. The full channel vector from all *N*_*t*_*K* transmit antennas to UE *u* is defined as:

$${𝐡}_{u}=[{𝐡}_{u,1}^{T},\ldots ,{𝐡}_{u,N_{t}}^{T}{]}^{T}.$$ (4)

The path loss is modeled as:

$$\text{PL}[\text{dB}]=32.4+20\log_{10}(f[\text{MHz}])+10\eta \log_{10}(d[\tau ]),$$ (5)

where η=3.5 is the path loss exponent. For *f* = 3500 MHz and *d* = 50 m, the path loss is approximately 91.5 dB. Shadow fading is modeled as a log-normal distribution with 8 dB standard deviation. Minimum mean square error (MMSE) channel estimation is performed uplink pilot symbols.

### 3.3 Sensing channel model

A line-of-sight (LOS) channel is considered between the APs and a target located at $\left(x_{t},y_{t}\right)$, with azimuth angle *ϕ* and elevation angle ϑ. The antenna array response vector is given by:

$$𝐚(\phi ,\vartheta )={\left[1,e^{j\pi \sin(\phi )\cos(\vartheta )},\ldots ,e^{j(K-1)\pi \sin(\phi )\cos(\vartheta )}\right]}^{T}.$$ (6)

The composite channel from all transmit APs to the target is:

$${𝐡}_{0}=[\sqrt{\beta_{1}}{𝐚}^{T}(\phi_{1},\vartheta_{1}),\ldots ,\sqrt{\beta_{N_{t}}}{𝐚}^{T}(\phi_{N_{t}},\vartheta_{N_{t}}){]}^{T},$$ (7)

where $\beta_{q}$ denotes the path loss for the link from AP *q* to the target.

### 3.4 Interference channel model

The interference at the sensing receive APs consists of both LoS components from other APs and non-line-of-sight (NLoS) clutter. While known LoS and static NLoS paths from permanent structures are pre-estimated and canceled, dynamic NLoS paths from moving objects are modeled as unknown interference [32]. The unknown NLoS channel between transmit AP *q* and receive AP *r* is represented by ${𝐇}_{r,q}\in {\mathbb{C}}^{K\times K}$. Correlated Rayleigh fading channels are used for the NLoS channels which are modeled using Kronecker model.

$${𝐇}_{r,q}={𝐑}_{rx,(r,q)}^{1/2}{𝐖}_{r,q}({𝐑}_{tx,(r,q)}^{1/2}{)}^{T},$$ (8)

where ${𝐖}_{r,q}$ has independent and identically distributed (i.i.d.) CN(0,1) entries, and ${𝐑}_{tx,(r,q)}$, ${𝐑}_{rx,(r,q)}$ are spatial correlation matrices with correlation coefficient α=0.7.

## 4 Localization and target detection

### 4.1 Localization

This section presents the localization techniques to estimate the position of a user node [33]. Let the source node whose location is to be estimated be the target node and the other *M* sensors that collect the measurements be the receivers.

A widely adopted technique is *Time of Arrival* (TOA), which measures the propagation delay between the target and each receiver. Provided the target and receivers are time-synchronized, multiplying the delay by the signal’s propagation speed yields the target-receiver distance. These distances enable multilateration methods to estimate the target’s position [34]. However, maintaining precise synchronization between a mobile target and multiple receivers is often impractical. To address this, *Time Difference of Arrival* (TDOA) methods are employed, wherein only the receivers must share a common time reference. By analyzing differences in signal arrival times, hyperbolic constraints are formed whose intersection yields the target’s estimated location. TDOA techniques are particularly effective for real-time localization in dynamic environments [35]. When receiver nodes are equipped with antenna arrays, they can estimate the *Direction of Arrival* (DOA) of the received signal. These angular measurements, combined with the known receiver positions, facilitate geometric triangulation, even in environments affected by multipath propagation. Modern localization systems often adopt *hybrid approaches* that integrate multiple modalities such as TOA, TDOA, DOA, Received Signal Strength (RSS), inertial sensor data, and heterogeneous radio technologies. Such fusion strategies mitigate the limitations of individual techniques, enhance robustness in NLoS conditions, and improve localization accuracy in complex environments.

The problem of localizing a target node in a two-dimensional Euclidean space ${\mathbb{R}}^{2}$ using TOA, TDOA and DOA measurements from *M* spatially distributed receivers is addressed next. Let the unknown position of the target be (*x*,*y*), and the known location of the *m*^*th*^ receiver be $\left(x_{m},y_{m}\right)$, where m=1,2,…,M.

### 4.2 TOA-based localization

Assuming perfect synchronization and LoS propagation, if the target transmits a signal at a known reference time *t* = 0, the TOA at the *m*^*th*^ receiver is given by:

$$t_{m}=\frac{\sqrt{(x_{m}-x{)}^{2}+(y_{m}-y{)}^{2}}}{c},$$ (9)

where *c* denotes the speed of light. The propagation distance is:

$$d_{m}=c\cdot t_{m}=\sqrt{(x_{m}-x{)}^{2}+(y_{m}-y{)}^{2}}.$$ (10)

This implies that the target lies on a circle centered at $\left(x_{m},y_{m}\right)$ with radius *d*_*m*_. With at least three such distances (M≥3), the target’s position can be uniquely determined via trilateration under ideal conditions [36].

In practice, TOA measurements are corrupted by noise, multipath effects, and bandwidth limitations. The noisy measurement is modeled as:

$$r_{m}=d_{m}+n_{m}=\sqrt{(x_{m}-x{)}^{2}+(y_{m}-y{)}^{2}}+n_{m},$$ (11)

where $n_{m}\sim 𝒩(0,\sigma_{d}^{2})$ represents additive white Gaussian noise (AWGN).

Each measurement defines an annular region around each receiver, and the intersection of these annuli provides an estimate of the target’s location. Estimation techniques such as Least Squares (LS), Maximum Likelihood (ML), or Bayesian filters are employed to refine localization [37].

Confidence intervals for the measured distance can be constructed as:

$$d_{m}\in [r_{m}-\gamma \sigma_{d},\;r_{m}+\gamma \sigma_{d}],$$ (12)

where *γ* is a confidence multiplier.

*Computational complexity analysis.*

Let *M* be the number of receivers and *L* be the number of signal samples. TOA localization involves estimation of signal arrival times followed by position estimation. At each of the *M* receivers, correlation or matched filtering is used for the TOA estimation over *L* received samples, resulting in a complexity of 𝒪(ML). Once the propagation delays are estimated, the target position is obtained using trilateration through LS or ML estimation. This involves solving a low-dimensional nonlinear optimization problem which incurs a computational cost of 𝒪(IM), where *I* denotes the number of iterations required for convergence. Therefore, the overall computational complexity of the TOA-based localization approach scales as 𝒪(ML+IM).

### 4.3 TDOA-based localization

TDOA methods eliminate the need for target-receiver synchronization by requiring synchronization only among the receivers. The arrival time at the *m*^*th*^ receiver is:

$$t_{m}=\frac{\sqrt{(x_{m}-x{)}^{2}+(y_{m}-y{)}^{2}}}{c}+\delta ,$$ (13)

where *δ* denotes the unknown transmission time offset.

Using receiver 1 as the reference, the TDOA is:


$$t_{m,1}=t_{m}-t_{1}$$

$$=\frac{\sqrt{(x_{m}-x{)}^{2}+(y_{m}-y{)}^{2}}-\sqrt{(x_{1}-x{)}^{2}+(y_{1}-y{)}^{2}}}{c}.$$ (14)

Multiplying by *c* yields the range difference:


$$d_{m,1}=ct_{m,1}$$

$$=\sqrt{(x_{m}-x{)}^{2}+(y_{m}-y{)}^{2}}-\sqrt{(x_{1}-x{)}^{2}+(y_{1}-y{)}^{2}}.$$ (15)

Each such equation defines a hyperbola with foci at receivers *m* and 1. The noisy range difference measurement is:

$$r_{m,1}=d_{m,1}+n_{m,1},$$ (16)

where $n_{m,1}=n_{m}-n_{1}\sim 𝒩(0,2\sigma_{d}^{2})$.

Define the measurement vector:

$$𝐫=[r_{2,1},r_{3,1},\ldots ,r_{M,1}{]}^{T},$$ (17)

and the theoretical range difference vector:

$$\bar{𝐝}(x,y)=[d_{2,1}(x,y),d_{3,1}(x,y),\ldots ,d_{M,1}(x,y){]}^{T}.$$ (18)

The observation model is:

$$𝐫\sim 𝒩(\bar{𝐝}(x,y),𝐂),$$ (19)

where the covariance matrix $𝐂\in {\mathbb{R}}^{\left(M-1)\times (M-1\right)}$ has:

- Diagonal entries: $C_{m-1,m-1}=2\sigma_{d}^{2}$,
- Off-diagonal entries: $C_{m-1,i-1}=\sigma_{d}^{2}$ for m≠i.

Thus,

$$𝐂=\sigma_{d}^{2}[\begin{array}{cccc} 2 & 1 & \ldots & 1\\ 1 & 2 & \ldots & 1\\ \vdots & \vdots & \ddots & \vdots \\ 1 & 1 & \ldots & 2 \end{array}].$$ (20)

The ML estimate of the target coordinates are obtained as

$$(\hat{x},\hat{y})=\arg\min_{\left(x,y\right)}(𝐫-\bar{𝐝}(x,y){)}^{T}{𝐂}^{-1}(𝐫-\bar{𝐝}(x,y)).$$ (21)

This non-convex optimization problem is typically solved via iterative algorithms such as gradient descent, Gauss-Newton, or expectation-maximization, requiring careful initialization.

Localization accuracy is assessed using the *Root Mean Square Error* (RMSE) given by:

$$RMSE=𝔼\left[(x-\hat{x}{)}^{2}+(y-\hat{y}{)}^{2}\right].$$ (22)

*Computational complexity analysis.*

In the TDOA-based localization approach, the computational complexity is mainly influenced by the estimation of time differences and the nonlinear position estimation process. The TDOA measurements are obtained through cross-correlation of the received signals across *M* synchronized receivers, resulting in a computational cost of 𝒪(ML), where *L* denotes the number of signal samples. The subsequent localization is formulated as an ML or weighted LS problem involving the inversion of an (M−1)×(M−1) covariance matrix and iterative optimization, which incurs a complexity of $𝒪(M^{3}\;+\;IM^{2})$, with *I* denoting the number of iterations. Consequently, the overall computational complexity of the TDOA-based localization method scales as $𝒪(ML+M^{3}\;+\;IM^{2})$.

### 4.4 DOA-based localization

*Direction-of-Arrival* (DOA), also known as *Angle-of-Arrival* (AOA), localization estimates the position of a target using angular information measured at multiple sensor nodes [38]. In a two-dimensional space, for *M* receivers positioned at known coordinates $\left(x_{m},y_{m}\right)$, and a target located at unknown coordinates (*x*,*y*), the DOA at the *m*-th receiver is:

$$\phi_{m}=\arctan\left(\frac{y-y_{m}}{x-x_{m}}\right),\;m=1,\ldots ,M.$$ (23)

Each measured angle defines a line extending from each receiver. The intersection point of at least two such lines (assuming non-collinear receivers) provides the target’s estimated location through triangulation [39]. In practice, DOA measurements are corrupted by angular noise, modeled as:

$$r_{m}=\phi_{m}+n_{m},\;n_{m}\sim 𝒩(0,\sigma_{\phi }^{2}).$$ (24)

The complete observation model is expressed as:

$$𝐫=\phi (x,y)+𝐧,\;𝐧\sim 𝒩(0,\sigma_{\phi }^{2}{𝐈}_{M}),$$ (25)

where ϕ(x,y) is the theoretical DOA vector.

The Maximum Likelihood (ML) estimate is obtained by minimizing the Mahalanobis distance:

$$(\hat{x},\hat{y})=\arg\min_{\left(x,y\right)}{\left(𝐫-\phi (x,y)\right)}^{T}{𝐂}^{-1}\left(𝐫-\phi (x,y)\right),$$ (26)

where $𝐂=\sigma_{\phi }^{2}{𝐈}_{M}$.

Due to the nonlinearity of the arctan(·) function, the estimation problem is non-convex and typically solved using grid search or iterative optimization algorithms.

Localization accuracy improves with:

- Reduced angular noise variance $\sigma_{\phi }^{2}$,
- Greater spatial diversity in receiver placement.

In three-dimensional scenarios, both azimuth and elevation angles are required. Systems using uniform linear arrays (ULAs) provide only azimuthal data, necessitating at least three receivers for localization due to conical ambiguity.

DOA techniques eliminate synchronization requirements but require multi-antenna arrays, making them hardware-intensive. When combined with TOA or TDOA methods, DOA enhances overall localization accuracy and robustness.

*Computational complexity analysis.*

In the DOA-based localization approach, the computational complexity is dominated by the estimation of the direction of arrival using antenna array processing and the subsequent geometric localization. For subspace-based DOA estimators, the computation of the sample covariance matrix using *K* antenna elements and *L* samples requires $𝒪(K^{2}L)$ operations, followed by an eigenvalue decomposition (EVD) with complexity $𝒪(K^{3})$. Additionally, angular search over *G* points incurs a complexity of $𝒪(GK^{2})$. Once the DOA estimates are obtained, the target position is determined via triangulation or ML estimation with negligible overhead compared to array processing. Therefore, the overall computational complexity of the DOA-based localization approach scales as $𝒪(K^{2}L+K^{3}+GK^{2})$.

### 4.5 Target detection

Target detection is a fundamental task in radar-based sensing systems, where the objective is to determine the presence or absence of an object at a specific location. Unlike localization methods in which the target actively transmits, radar-based detection relies on transmitting known waveforms and analyzing their reflections from passive targets.

A key parameter governing detectability is the radar cross section (RCS) $\sigma_{RCS}$ which characterizes how strongly a target reflects incident electromagnetic energy and is modeled using Swerling models. Consider *L* complex baseband samples *y*[1],...,*y*[*L*] collected during a single detection interval. Under the Swerling 1 model the target’s complex radar-cross-section (RCS) coefficient $\chi_{RCS}$ remains constant across the *L* samples, such that $\chi_{RCS}\sim 𝒞𝒩(0,1)$. While in Swerling 2 model, the target’s RCS fluctuate rapidly such that at each symbol time it takes a new realization as $\chi_{RCS}[l]\sim 𝒞𝒩(0,1)$. Denoting the additive receiver noise samples by n[l]~𝒞𝒩(0,1) and average received power by *P*_*R*_, the binary hypotheses in model-1 becomes

$$H_{0}:\;y[l]=n[l],l=1,2...L,\;H_{1}:\;y[l]=\sqrt{P_{R}}\chi_{RCS}+n[l],l=1,2...L.$$ (27)

For a given false alarm probability $P_{F}=\alpha$, the detection probability can be maximized using Neyman-Pearson detector.

$$P_{F}=\exp\left(-\frac{\gamma }{L\sigma^{2}}\right)$$ (28)

where *γ* is the detection threshold, *L* is the number of samples, and $\sigma^{2}$ is the noise variance. For a desired false-alarm level *α*, the threshold is set as

$$\gamma =-L\sigma^{2}\ln(\alpha )$$ (29)

With effective SNR $\rho =\frac{P_{R}L}{\sigma^{2}}$, normalized threshold τ=−ln(α), the closed form of detection probability is

$$P_{D}=\frac{e^{-\tau /(1+\rho )}}{\left(1+\rho \right)}\sum_{k=0}^{L-1}\frac{1}{k!}{\left(\frac{\tau \rho }{1+\rho }\right)}^{k}$$ (30)

## 5 Results and discussion

The proposed ISAC system model defined in Sect 2 is modeled in MATLAB and the results are presented here in this section. The simulation setup is run for 10^4^ iterations and the average is taken to get the results. The key parameters used in the simulation of proposed ISAC system model and channel configuration are summarized in Table 3 below.

**Table 3:** Considered parameters for the simulation.

Parameter	Value
Number of Transmit APs ($N_{\text{t}}$)	4
Number of Receive APs (*M*)	4
Antennas per AP (*K*)	8
Number of UEs (*U*)	6
Bandwidth	20 MHz
Carrier Frequency (*f*)	3.5 GHz
Max Transmit Power per AP (*P*_*tx*_)	30 dBm (1 W)
Path loss exponent (*η*)	3.5
Shadow Fading Std. Dev.	8 dB
Channel Coherence Time	2 ms
Sensing Azimuth/Elevation	$45^{\circ }/30^{\circ }$
Spatial Correlation Factor (*α*)	0.7

**Fig 2:**
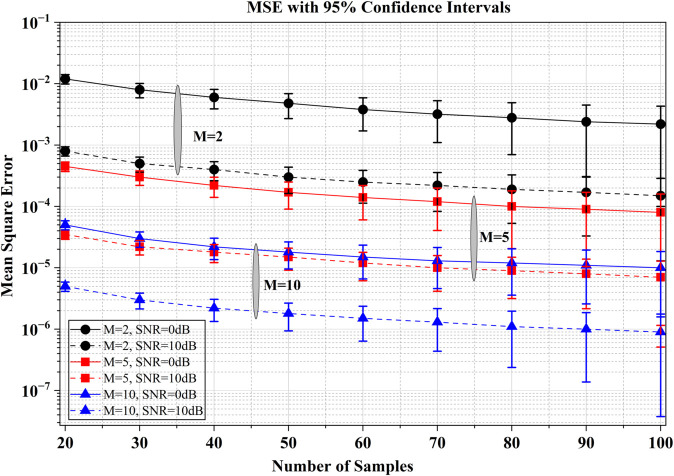
MSE performance of DOA estimation as a function of number of samples (L) for different *M* and SNR.

Fig 2 presents the variation of MSE with respect to the number of samples *L* for different values of *M* and SNR. It can be observed that the MSE decreases as the number of samples increases, indicating improved estimation accuracy with more data. Furthermore, for a fixed number of samples, the MSE reduces as both *M* and SNR increase. At SNR of 10 dB and *L*=60, doubling *M* (from 5 to 10) yields MSE of $2\;\times \;10^{-5}$ from $1.5\;\times \;10^{-4}$ offering a gain of 8.75dB. This demonstrates that systems with a higher number of cooperative receivers and better signal quality achieve more accurate estimations. Specifically, configurations with *M* = 10 consistently exhibit the lowest MSE, followed by *M* = 5 and *M* = 2. The downward trend of the curves confirms that increasing the number of samples and receivers enhances the reliability of the estimation process, while higher SNR values further suppress the estimation error. Overall, the figure highlights the combined influence of sample size, receiver diversity, and signal strength in minimizing estimation errors and improving system performance.

**Fig 3:**
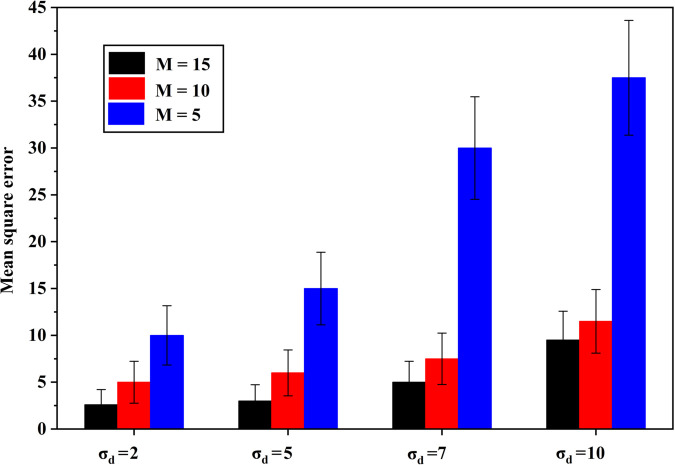
Variation of MSE of TDOA estimation for different noise standard deviation $\sigma_{d}$ under different *M*.

The graph shown in Fig 3 depicts the variation of MSE with respect to different noise standard deviations ($\sigma_{d}$) under various values of *M*. It can be observed that as the noise standard deviation increases, the MSE also increases for all values of *M*. Furthermore, the rate of increase in MSE is higher for smaller *M* values, indicating that systems with a lower *M* are more sensitive to noise. Conversely, higher *M* values exhibit relatively lower MSE, signifying better robustness against noise. This demonstrates that increasing *M* enhances estimation accuracy by reducing the impact of noise on the system performance.

Table 4 gives the localization RMSE of TDOA and DOA estimation for varying values of *M*. The localization error decreases with increasing *M* in both estimation methods. For varied values of noise standard deviation $\sigma_{d}$ (in TDOA estimation) and $\sigma_{\phi }$ (in DOA estimation), the RMSE values are obtained and tabulated.

**Table 4:** Localization RMSE (m) for different values of $\sigma_{d}$ and $\sigma_{\phi }$ for varied *M*.

M	TDOA Estimation		DOA Estimation	
	$\sigma_{d}$ **= 10**	$\sigma_{d}$ **= 5**	$\sigma_{\phi }$ **= 6**	$\sigma_{\phi }$ **= 3**
5	38 m	15 m	13 m	6.5 m
10	12 m	6 m	10 m	5 m
15	10 m	4 m	8 m	4.5 m
20	8 m	3.5 m	7 m	4 m
25	7 m	3 m	6 m	3.9 m

**Fig 4:**
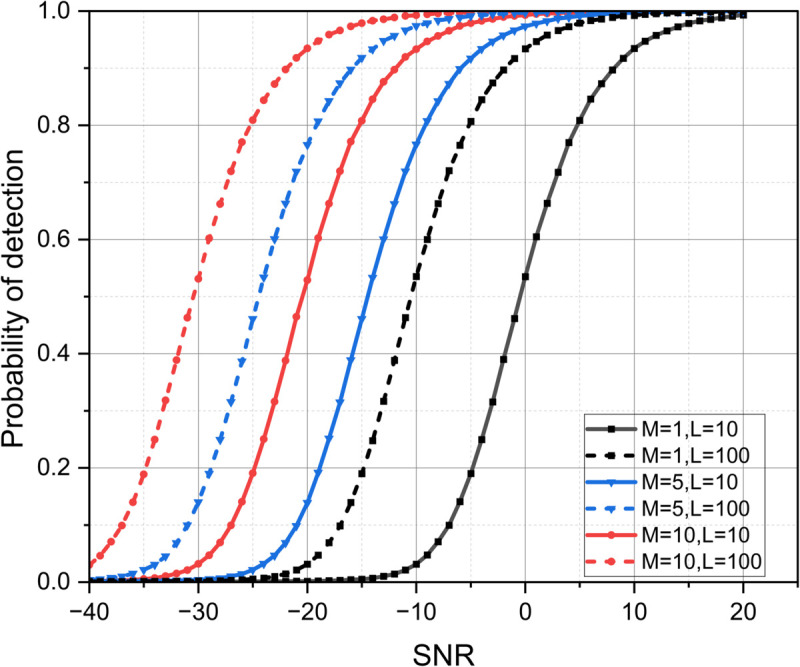
Variation of *P*_*D*_ with SNR for different *M* and *L* using Swerling model-1.

Fig 4 illustrates the variation of probability of detection *P*_*D*_ with SNR for different combinations of the number of receivers (*M*) and observation lengths (*L*) with model-1. It can be observed that *P*_*D*_ improves with an increase in SNR for all combinations. However, systems with higher values of *M* and *L* exhibit a noticeable improvement in detection performance, as their curves shift toward the lower SNR region. This indicates that reliable detection can be achieved even under low SNR conditions when the number of cooperative receivers and observation intervals is increased. In contrast, smaller values of *M* and *L* require higher SNR levels to reach similar detection probabilities. It is observed that in order to achieve *P*_*D*_=1, there is an SNR gain of 15 dB with *M*=10, *L*=10 over *M*=1 and *L*=10. Overall, the figure confirms that increasing the number of receivers and observation samples enhances estimation accuracy and detection reliability, emphasizing the effectiveness of cooperative sensing in improving system performance under noisy environments.

**Fig 5:**
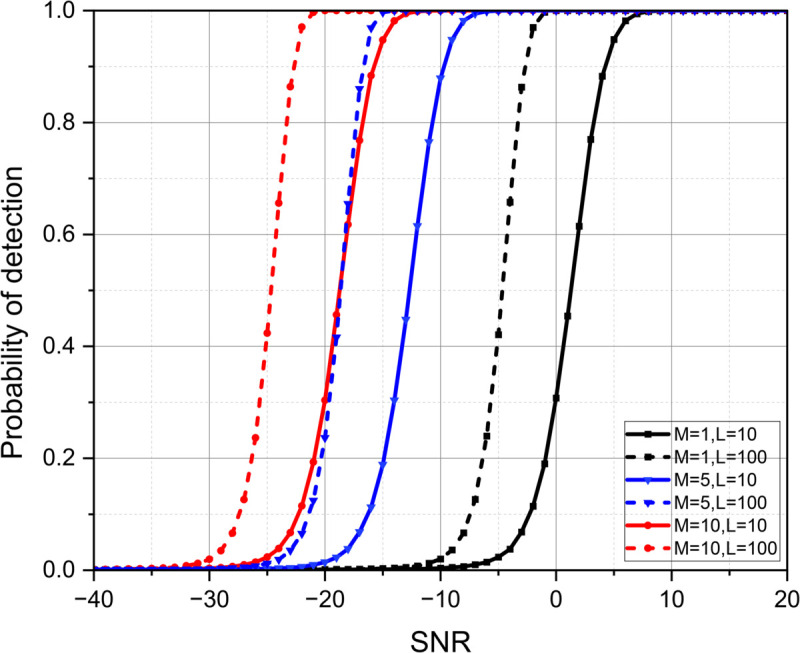
Probability of detection versus SNR with Swerling model-2 for different (*M*) and (*L*).

Fig 5 illustrates the variation of *P*_*D*_ with SNR for model-2 for different *M* and *L*. It is observed that as the SNR increases, the probability of detection improves across all configurations. Moreover, systems with a higher number of antennas (*M*) achieve better detection performance due to enhanced spatial diversity and signal combining gain. Similarly, a longer sequence length (*L*) enhances detection reliability by averaging out noise effects. There is a left shift of 20 dB in *P*_*D*_ curve when number of receivers *M* changes from 1 to 10. Among all cases, the configuration with both higher *M* and *L* values (e.g., M=10,L=100) provides the best performance, achieving a high probability of detection even at lower SNR levels. Tables 5 and 6 present the comparison of proposed approach with existing baseline approaches for localization RMSE and probability of detection *P*_*D*_ respectively. Three baseline approaches are considered for comparison, namely conventional communication-centric ISAC design, sensing-centric ISAC design and non-cooperative ISAC design approach. The localization RMSE and *P*_*D*_ are evaluated for low, medium and high SNR ranges.

**Table 5:** Comparison of proposed approach with existing baseline approaches for localization RMSE.

Ref	Scheme	Localization RMSE		
		Low SNR (–5 dB)	Medium SNR (5 dB)	High SNR (15 dB)
[13]	Communication-centric ISAC	42 m	21 m	11 m
[14]	Sensing-centric ISAC	31 m	15 m	7.8 m
[21]	Non-cooperative ISAC	26 m	12 m	7.8 m
This work	Proposed C-RAN ISAC	18 m	6.5 m	3.9 m

**Table 6:** Comparison of proposed approach with existing baseline approaches for probability of detection *P*_*D*_.

Ref	Scheme	Probability of Detection *P*_*D*_		
		Low SNR (–5 dB)	Medium SNR (5 dB)	High SNR (15 dB)
[13]	Communication-centric ISAC	0.38	0.71	0.92
[14]	Sensing-centric ISAC	0.52	0.84	0.97
[21]	Non-cooperative ISAC	0.58	0.88	0.98
This work	Proposed C-RAN ISAC	0.75	0.96	≈1.00

The proposed ISAC approach consistently achieves lower localization RMSE than the baseline approaches. For target detection, the proposed method provides a left-shift of up to 8-12 dB in SNR for a given *P*_*D*_ owing to cooperative multi-static sensing and joint processing.

## 6 Deployment perspectives and future directions

### 6.1 Integration with edge AI and 6G digital twin

From a deployment perspective, the proposed centralized ISAC framework can be extended by integrating edge AI and 6G digital twin technologies.

- Edge intelligence deployed at the C-RAN edge cloud can process sensing and communication data in real time, enabling learning-assisted localization, target detection, beam management and environment awareness under mobility and NLoS conditions. This significantly reduces latency and fronthaul overhead while enhancing system responsiveness in dense and dynamic environments [40].
- 6G digital twin can maintain a virtual representation of the physical environment, network topology and target dynamics by continuously ingesting sensing data from ISAC nodes [41]. This enables predictive sensing, proactive resource allocation and performance optimization without disrupting live network operations, facilitating self-configuration and self-healing capabilities [42].
  Such a synergy between ISAC, edge AI and digital twins aligns with the vision of intelligent, self-aware, and autonomous 6G networks and further enhances the practical applicability of the proposed framework.

### 6.2 Future directions

Future work focuses on addressing key practical challenges in ISAC-based 6G networks, including accurate 3D localization, reliable operation under high mobility scenarios and device synchronization issues.

- 3D localization: The localization model can be extended to 3D positioning for applications such as aerial vehicles, drone-assisted sensing, and indoor–outdoor integrated networks. To achieve high-precision 3D localization under multipath conditions, future work will focus on joint estimation of azimuth and elevation angles, elevation-aware beamforming, exploitation of planar or 3D antenna arrays and multi-node ISAC cooperation in complex NLoS environments [43].
- High mobility scenarios: Fast channel variations, severe Doppler effects in high-mobility scenarios and beam misalignment can degrade both communication and sensing accuracy [44]. Future work may incorporate mobility-aware beam tracking, Doppler-resilient waveforms and learning-assisted prediction at the edge to enable robust localization and target detection in vehicular and aerial environments.
- Device synchronization issues: The fundamental limitation particularly for TOA- and TDOA-based localization is the device synchronization. Practical deployments must address clock offsets, phase noise and hardware impairments across distributed access points [45]. Future research will explore synchronization-free localization techniques, joint clock-offset estimation, cooperative timing alignment and sensing-assisted synchronization to mitigate these effects.

## 7 Conclusion

To manage the spectrum and hardware overhead in 6G networks owing to increasing data traffic and device density, this work presents an ISAC framework emphasizing the joint design of sensing and communication within a C-RAN architecture. The proposed system employs multiple transmit and receive APs with uniform linear antenna arrays, enabling simultaneous data transmission and environmental sensing. The localization in ISAC framework is analyzed through TOA, TDOA and DOA estimation techniques, while target detection is performed using Swerling models. The localization MSE and *P*_*D*_ is evaluated for different *M*, *L* and SNR values under different noise levels. It is observed that for a fixed *L*, the MSE reduces as both *M* and SNR increase. At SNR of 10 dB and *L*=60, doubling *M* (from 5 to 10) yields MSE of $2\;\times \;10^{-5}$ from $1.5\;\times \;10^{-4}$ offering a gain of 8.75dB. Further, for varied values of noise standard deviation $\sigma_{d}$ (in TDOA estimation) and $\sigma_{\phi }$ (in DOA estimation), the RMSE values are obtained. The target detection performance using Swerling models show that *P*_*D*_ improves with an increase in SNR for all combinations of *M* and *L*. Higher *M* achieves better detection performance due to enhanced spatial diversity and signal combining gain. Similarly, a longer *L* enhances detection reliability by averaging out noise effects. There is a left shift of 20 dB in *P*_*D*_ curve when *M* changes from 1 to 10. These findings validate that the proposed ISAC framework significantly enhances localization precision, spectral efficiency, and energy utilization. Overall, this study establishes a foundation for developing intelligent, spectrum-efficient, and environment-aware 6G networks, paving the way for future applications in autonomous systems, smart cities, and large-scale IoT infrastructures.
